# Genomic and transcriptomic analysis suggests potential regulatory relationships among *MC1R*, *TUBB3*, and *PMEL* associated with black-flecked plumage in chickens

**DOI:** 10.1186/s12864-026-13107-w

**Published:** 2026-06-29

**Authors:** Yifan Guo, Yuqi Chen, Huanjie Guo, Binghui Wang, Yanzhang Gong, Jingyi Li

**Affiliations:** Key Laboratory of Agricultural Animal Genetics, Breeding and Reproduction of Ministry of Education, College of Animal Science and Technology and College of Veterinary Medicine, Huazhong Agricultural University, Wuhan, 430070 PR China

**Keywords:** Chicken, Dominant white, Genomic, *MC1R*, Transcriptomic, *TUBB3*

## Abstract

The *Dominant white* (*I*) allele in chickens suppresses the expression of black eumelanin, resulting in white plumage. However, both previous reports and our observations showed that some heterozygous (*I*/*i*^*+*^) individuals exhibited black-flecked plumage rather than completely white plumage, unlike homozygous dominant white chickens. This phenomenon suggests incomplete penetrance and incomplete dominance of the *I* allele. The genetic mechanisms underlying the phenotypic divergences in *I*/*i*^*+*^ individuals remain unclear.

In this study, through integrated genomic and transcriptomic analyses of chickens with different *I* locus genotypes and feather color phenotypes, we identified candidate variants in *MC1R* (p.T143A) and *TUBB3* (p.R202R) that may be associated with phenotypic variation in pigmentation. Notably, *PMEL* serves as the primary causal gene underlying the Dominant white phenotype, while the identified loci may act as modifying factors influencing residual melanin deposition in heterozygous individuals. Furthermore, our data suggest that biological processes such as glial cell formation and development, as well as responses to organic cyclic compounds, may participate in regulating the spatiotemporal distribution of melanin, thereby influencing the formation of black-flecked feathers. This finding provides a new perspective on the molecular mechanisms of incomplete penetrance and incomplete dominance of *I* and reveals the complexity of the melanin deposition regulatory network.

**Supplementary Information:**

The online version contains supplementary material available at https://doi.org/10.1186/s12864-026-13107-w.

## Introduction

**Fig. 1:**
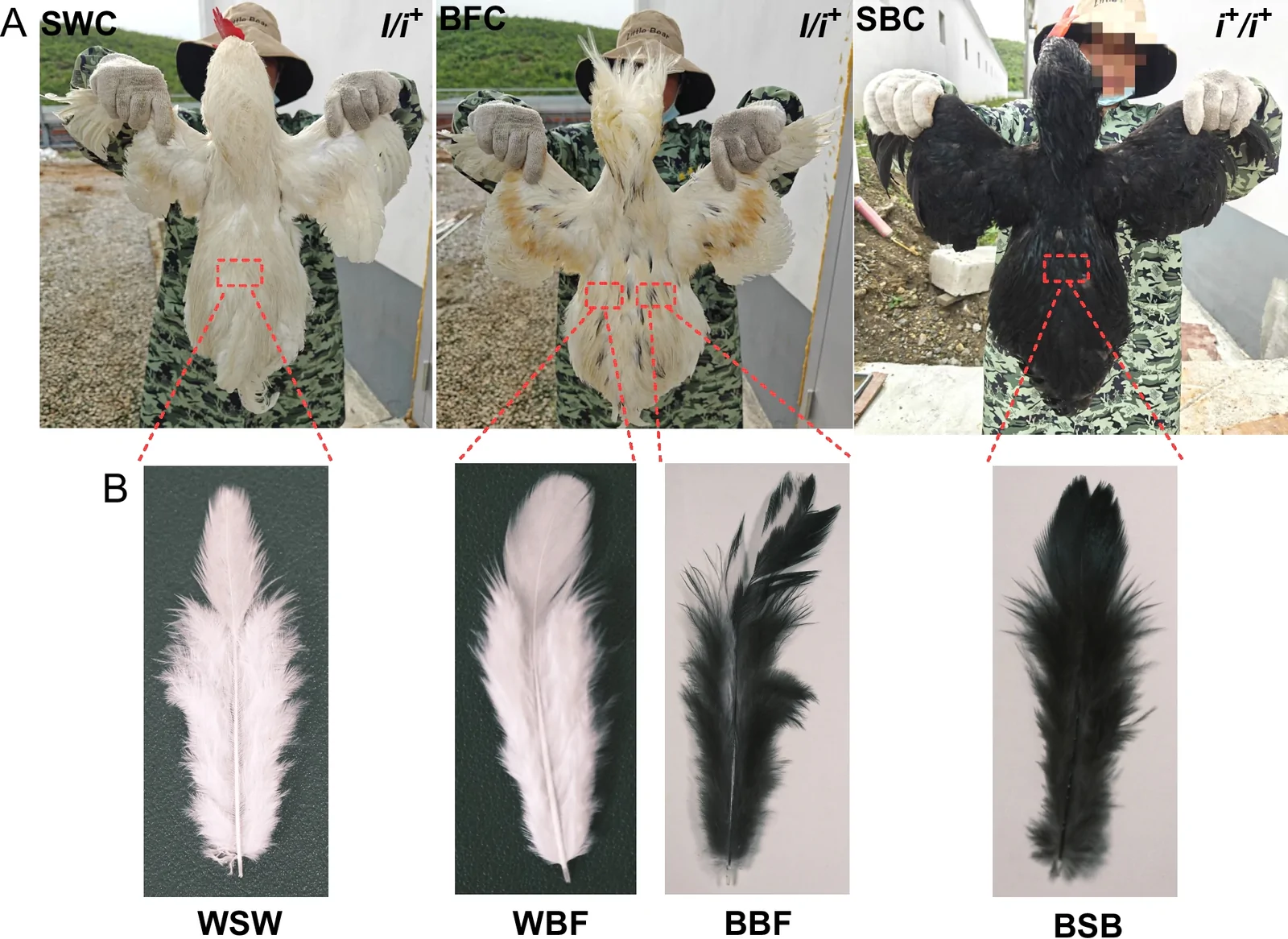
Examples of chickens with different plumage colors and the sampling of feather follicles. **A** Photos of chickens with different plumage colors used for whole-genome resequencing. **B** Schematic representation of the feather follicle sampling sites used for RNA-seq. Photo by: Yuqi Chen

Birds exhibit extremely diverse feather coloration in nature, ranging from pigment-based colors such as black and white to structural colors produced by the microstructure of feathers [1]. These colors not only play crucial roles in avian adaptation but also capture considerable human preference. White plumage is widely adopted in modern layer and broiler production, as unpigmented feathers improve the visual appeal of processed carcasses [2]. The *Dominant white *mutation (*I*) has played a significant role in the industries of white layers and even broilers, and in fact represents one of the earliest traits studied after Mendel’s foundational work in genetics [3]. Kerje et al. reported that *I* is closely associated with a 9-bp insertion in the transmembrane region of *PMEL* [4]. *PMEL* encodes a type I single-pass transmembrane glycoprotein, promotes melanosome maturation by assembling into striated amyloid fibrils during stage II melanosomes, extending the organelles and providing scaffolds for eumelanin deposition [5, 6]. Eumelanin and pheomelanin are the two types of melanin and are the key determinants of feather color in chickens [7–9]. Eumelanin ranges from deep black to gray, manifesting in feathers as black or gray plumage, whereas pheomelanin exhibits yellow to red hues, producing red or yellow feathers [10]. The switching between eumelanin and pheomelanin in chicken feathers is mainly regulated by *MC1R* [11]. At least six *MC1R* alleles with different phenotypes have been reported in chickens [12], listed in descending order of eumelanin deposition: extended black (*E*), birchen (*E*^*R*^), wild type (*e*^+^), brown (*e*^*b*^), gold-laced feather (*e*^*bc*^), and wheaten (*e*^*wh*^ or *e*^*y*^). These alleles regulate the distribution of eumelanin in the plumage. When a chicken carries the *E* allele, its feathers are filled with eumelanin but lack pheomelanin [13]. Under this genetic background, the 9-bp insertion in *PMEL* inhibits eumelanin synthesis, resulting in completely white plumage in White Leghorn individuals [4]. However, in breeding practices, when selecting against *I* via marker-assisted selection, we observed that heterozygotes (*I*/*i*^*+*^) could exhibit two distinct phenotypes: some individuals showed complete dominance (solid white, Fig. 1), while others displayed incomplete dominance, with scattered black spots appearing on white feathers (black-flecked plumage, Fig. 1). Similar phenotypic observations have been reported previously [4, 13]. This suggests the presence of unknown mutations that modulate the inhibitory effect of *I* on eumelanin, leading to incomplete penetrance, i.e., phenotypic divergence in heterozygotes.

Based on these observations, we hypothesized that additional genetic factors and local regulatory mechanisms may modulate the inhibitory effect of the *Dominant white* allele on eumelanin deposition, thereby contributing to the phenotypic divergence observed among heterozygous (*I*/*i*^*+*^) individuals. Therefore, the objective of this study was to identify candidate genomic regions, genes, and transcriptomic features associated with black-flecked plumage formation under the *I*/*i*^+^ background. In this study, we generated whole-genome resequencing data from three phenotype-genotype combinations, solid white chickens (*I*/*i*^*+*^), black-flecked chickens (*I*/*i*^*+*^), and solid black chickens (*i*^*+*^/*i*^*+*^), as well as RNA-seq data from two types of white feather follicle tissues (from solid white and black-flecked chickens) and two types of black feather follicle tissues (from black-flecked and solid black chickens). Through integrative analysis, this study provides new insights into the genetic basis underlying phenotypic differentiation under the *I*/*i*^+^ background and offers a better understanding of the molecular mechanisms regulating plumage pigmentation. The identified genetic markers may also assist in marker-assisted selection of plumage color in chicken breeding.

## Material and methods

### Animals and data collection

A total of 64 hens were selected from a specialized layer line maintained at Hubei Shendi Agricultural Science and Trade Co., Ltd. (China). All individuals originated from the same breeding population and were raised under identical environmental and management conditions, thereby minimizing potential confounding effects caused by population structure or environmental variation. These chickens were assigned into three feather color phenotypes based on their appearance and genotype of *I* locus (as shown in Fig. 1A): solid white chickens (SWC, *I*/*i*^+^, *n* = 13), black-flecked chickens (BFC, *I*/*i*^+^, *n* = 18), and a control group without the effect of *I*: solid black chickens (SBC, *i*^+^/*i*^+^, *n* = 33). The amounts of SWC and BFC in the population is relative small since we required both certain phenotypes and genotype. After phenotypic recording, approximately 200 μL of blood was collected from the brachial vein of each of the 64 individuals and stored for subsequent genomic analyses.

Feather follicles were collected non-lethally from regenerated feathers following manual feather plucking. All chickens were from the same specialized layer line and were raised under identical environmental and nutritional conditions. The sampled individuals were adult, and all samples were collected from the dorsal feather tract to minimize body regional variation. Feather follicle tissues were collected during the active growth phase of feather regeneration, approximately 15 days after feather plucking, when plumage color could be clearly distinguished. For RNA-seq analysis, four types of feather follicle samples were obtained (Fig. 1B): white feathers from solid white chickens (WSW, *n* = 3), white feathers from black-flecked chickens (WBF, *n* = 3), black feathers from black-flecked chickens (BBF, *n* = 3), and black feathers from solid black chickens (BSB, *n* = 3). All tissue samples were immediately frozen in liquid nitrogen and then stored at − 80 °C until further processing.

No birds were sacrificed during sample collection, as all procedures were non-lethal and conducted under approved ethical guidelines.

### Genotyping of *I* locus

The *I* locus was genotyped using a PCR–RFLP approach. Primers used in this study were designed by the authors based on the *PMEL* reference sequence. We amplified genomic DNA with primer-F (GAGGTGGGAGAGGGATGG) and primer-R (AGTGCTGTGTTCTCCCCGTGT), generating a 296 bp fragment. PCR amplification was performed with an initial denaturation at 94 °C for 5 min, followed by 14 cycles of denaturation at 94 °C for 15 s, annealing at 68 °C for 15 s with a decrease of 1 °C per cycle, and extension at 72 °C for 30 s, then 25 cycles of denaturation at 94 °C for 15 s, annealing at 55 °C for 15 s, and extension at 72 °C for 30 s, with a final extension at 72 °C for 5 min. The PCR products were digested with the restriction enzyme BsrBI at 37 °C for 3 h and separated the resulting fragments by agarose gel electrophoresis. Genotypes were determined based on fragment size patterns: individuals of *i*^+^/*i*^+^ showed two bands of 223 bp and 66 bp; individuals of *I*/*I* showed three bands of 184 bp, 66 bp, and 46 bp; and *I*/*i*^+^ showed four bands of 223 bp, 184 bp, 66 bp, and 46 bp (Fig. S1).

### Genomic sequencing and sequence read alignment

Blood samples collected from the 64 individuals were used for genomic DNA extraction using the DNeasy Blood and Tissue Kit (QIAGEN). Library construction was performed with the YZSeqTn5 Library Prep Kit (Wuhan Yingzi Gene Technology Co., Ltd). The following quality control was conducted using the Fragment Analyzer 5300 (Agilent). Qualified libraries were sequenced with an average of 15 × coverage on a DNBSEQ-T7 platform (BGI) with a PE150 strategy. The sequencing was performed by Wuhan Yingzi Gene Technology Co., Ltd.

Raw sequencing reads were first processed using fastp v0.23.4 with the paremeters of -l 36 -n 5, to remove adapter sequences and low-quality reads [14]. The clean reads were aligned to the chicken reference genome (GRCg6a) using Burrows-Wheeler Aligner (BWA) v0.7.17 [15]. SAMtools v1.3.1 [16] was used to sort and remove duplicates. The results were subsequently realigned and recalibrated using GATK v3.8 to ensure high-quality mapped reads [17].

### RNA-seq data generation and read alignment

Feather follicle tissues collected during the active growth phase of feather regeneration were used for RNA sequencing. Total RNA was extracted from the follicles following the RNA extraction kit EASYspin Plus (Aidlab). The RNA sequencing was performed by Beijing Biomarker Technologies Co., Ltd.

Raw reads were first subjected to quality filtering using fastp v0.23.4 with the paremeters of -l 30 -n 15, to remove adapter sequences and low-quality bases [18]. The clean reads were then aligned to the chicken reference genome (GRCg6a) using STAR v2.7.9a with default mapping parameters [19]. Gene-level read counts were quantified using featureCounts v2.0.1 [20]. The resulting count matrix was used for downstream gene expression and differential expression analyses.

### SNP calling, quality control, and annotation

Variant detection for individual samples and merging of genotyping results across all samples was performed using Sentieon software [21] with parameters “–algo Haplotyper; –algo GVCFtyper”. The detected variants were stored in the standard Variant Call Format (VCF). Preliminary filtering of whole-genome resequencing data was conducted using GATK v3.8 [17] with thresholds “QD < 2.0 || FS > 60.0 || MQ < 40.0 || MQRankSum < −12.5 || ReadPosRankSum < −8.0”. Subsequent quality control was performed using PLINK v1.9 with a threshold of “–geno 0.05 –maf 0.01” [22]. Genotypes for candidate mutations were extracted from the final VCF file generated after variant calling and quality control. Genotypic frequencies in different groups were calculated based on individual genotype information from the VCF file.

In addition, WGS data from NCBI SRA database, consists of 68 red junglefowl (RJF) individuals (Table S1), were also analysed to genotype the candidate mutations, following the same alignment and SNP calling procedures.

### Genetic differentiation analysis

For pairwise comparisons among the three phenotypic groups, genetic differentiation was assessed using VCFtools v0.1.17 [23]. A sliding window approach was applied with a window size of 50 kb and a step size of 10 kb to calculate the average *F*_*ST*_ value for each window across the genome. The resulting *F*_*ST*_ values were then standardized to obtain *ZF*_*ST*_ values using the following formula:

$${{\boldsymbol{Z}}{\boldsymbol{F}}}_{{\boldsymbol{S}}{\boldsymbol{T}}{\boldsymbol{i}}}=\frac{{{\boldsymbol{F}}}_{{\boldsymbol{S}}{\boldsymbol{T}}{\boldsymbol{i}}}-{\boldsymbol{\mu}}}{{\boldsymbol{\sigma}}}$$

where *F*_*ST*i_ represents the *F*_*ST*_ value of the *i* th window, and *μ* and *σ* denote the genome-wide mean and standard deviation of all *F*_*ST*_ values, respectively. The windows with top 1% or 0.1% of genome-wide *ZF*_*ST*_ values were defined as candidate regions.

### Differential expression analysis

Gene expression profiling was performed based on raw read counts using DESeq2 v1.30.0 [24]. Normalization of read counts, estimation of dispersion, and statistical testing were carried out following the standard DESeq2 workflow. Differentially expressed genes (DEGs) were identified using Wald tests and adjusted using the Benjamini–Hochberg method [24, 25]. Genes with adjusted *P* < 0.05 and | log₂(fold change) |≥ 1 were considered significant [26–28]. The direction of differential expression was determined based on the sign of log₂(fold change) values. To reduce potential biases associated with a single enrichment tool, Gene Ontology (GO) enrichment analyses were performed using both the clusterProfiler package [29] and the g:Profiler web server [30], and the results were interpreted based on the combined enrichment patterns identified by the two approaches.

### Data visualization

The SNP density plot and manhattan plots were generated using the CMplot package in R v4.41 [31]. Linkage disequilibrium (LD) analysis was performed using LDBlockShow [32] to calculate and visualize pairwise LD based on the squared correlation coefficient (*r*^*2*^). LD heatmaps were generated using default settings of the software, and *r*^*2*^ values were displayed using the built-in color scale. For genomic principal component analysis (PCA), LD pruning was first conducted using PLINK v1.9 (–indep-pairwise 50 5 0.2), and PCA was subsequently performed using the –pca function in PLINK v1.9. For transcriptomic PCA [22], normalized gene expression values (FPKM) were used as input. PCA was performed using the FactoMineR and factoextra packages in R [33, 34], and visualized with color palettes provided by the RColorBrewer package [35]. Heatmaps of DEGs were generated using the pheatmap package [36]. Volcano plots were constructed using ggplot2 [37]. Bubble plots of enriched GO terms were visualized using the OmicStudio online tool [38].

### *MC1R* sequence analysis

*MC1R* protein sequences from representative vertebrate species were retrieved from the NCBI database (https://www.ncbi.nlm.nih.gov/). Multiple sequence alignment was performed using MAFFT v7.525 [39], and alignment results were visualized using ESPript 3.2 [40]. Representative species were selected to cover major vertebrate lineages for comparative analysis of the amino acid residue at position 143 of chicken.

### Public Hi-C data analysis

Publicly available chicken Hi-C data were obtained from the FAANG project reported by Pan et al. [41]. The Hi-C interaction map used in this study was generated from chicken liver tissue. These data were used only as a reference for higher-order chromatin organization and topologically associating domain (TAD) structure surrounding the *MC1R*-*TUBB3* locus.

## Results

### Genetic differentiation analysis of distinct phenotypes in *I*/*i*^+^ individuals

**Fig. 2:**
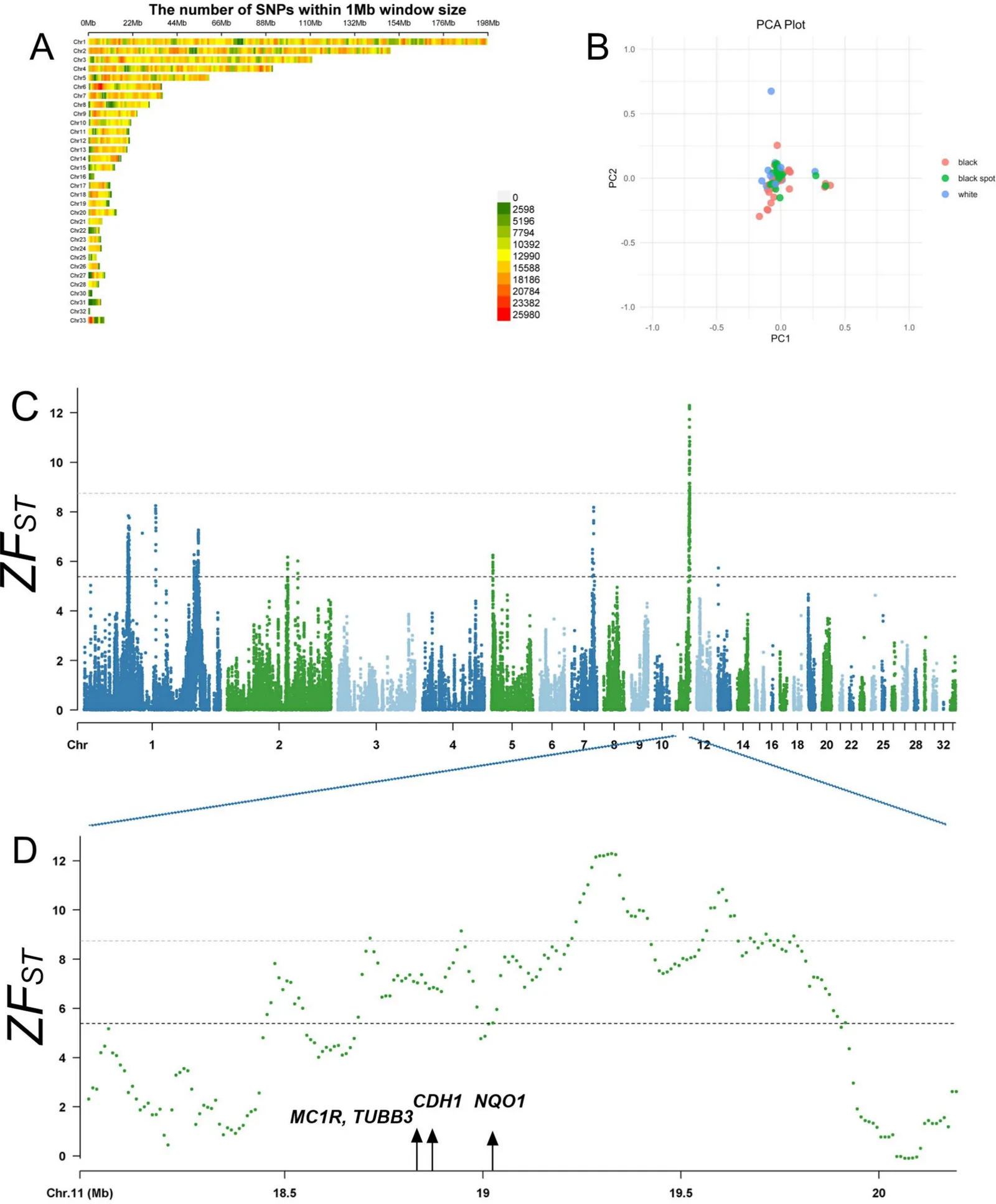
The genome-wide SNP distribution, population structure, and *ZF*_*ST*_ analysis of individuals with different feather color phenotypes. **A** SNP density plot based on WGS data of 64 individuals. **B** Principal Component Analysis (PCA) plot of 64 individuals. **C** Manhattan plot of *ZF*_*ST*_ analysis between *Dominant White* heterozygotes with solid white chicken and those with black-flecked chicken. The first gray threshold line represents the top 0.1% *ZF*_*ST*_ values, while the second black threshold line represents the top 1% *ZF*_*ST*_ values. **D** Manhattan plot of the candidate region on chromosome 11

After variant filtering and quality control, a total of 12,934,798 biallelic SNPs were identified across the 64 individuals (Fig. 2A). PCA revealed no clear genetic stratification among the SWC, BFC, and SBC groups (Fig. 2B), aligning with the fact that the individuals exhibiting the three feather-color phenotypes originated from the same population. They shared similar genetic background but differed primarily in feather pigmentation.

To identify genomic variation underlying the phenotypic divergence between SWC and BFC plumage in *I*/*i⁺* birds, we followed previous studies and calculated genome-wide *ZF*_*ST*_ values based on a sliding window approach to detect regions showing significant allelic frequency differentiation between phenotypically defined groups [42, 43]. Windows in the top 1% of the *ZF*_*ST*_ distribution were considered candidate regions. A pronounced peak signal was observed on chromosome 11. This candidate region harbored 78 genes, including *MC1R*, *TUBB3*, *PDF*, and *VPS4A* among 70 protein-coding genes (Fig. 2C, D, Table S2). In addition, elevated *ZF*_*ST*_ signals (top 1%) were also detected on chromosomes 1, 2, 5, and 7 (Fig. 2C).

Peak signal of the *ZF*_*ST*_ in the same region on chromosome 11 was also detected by the comparison between SWC and SBC, but not between BFC and SBC (Fig. S2A). Suggesting that the genetic element(s) contributing to the solid black chicken, is also responsible for the black-flecked feathers in the carriers of *I*. Besides, in both the comparisons between SWC and SBC, BFC and SBC, peak regions of *ZF*_*ST*_ on chromosome 33 including *PMEL* were detected (Fig. S2B), which is expected since the *PMEL* genotypes were known before the whole genome sequencing.

### Differences of expression profiles between black and white feathers

**Fig. 3:**
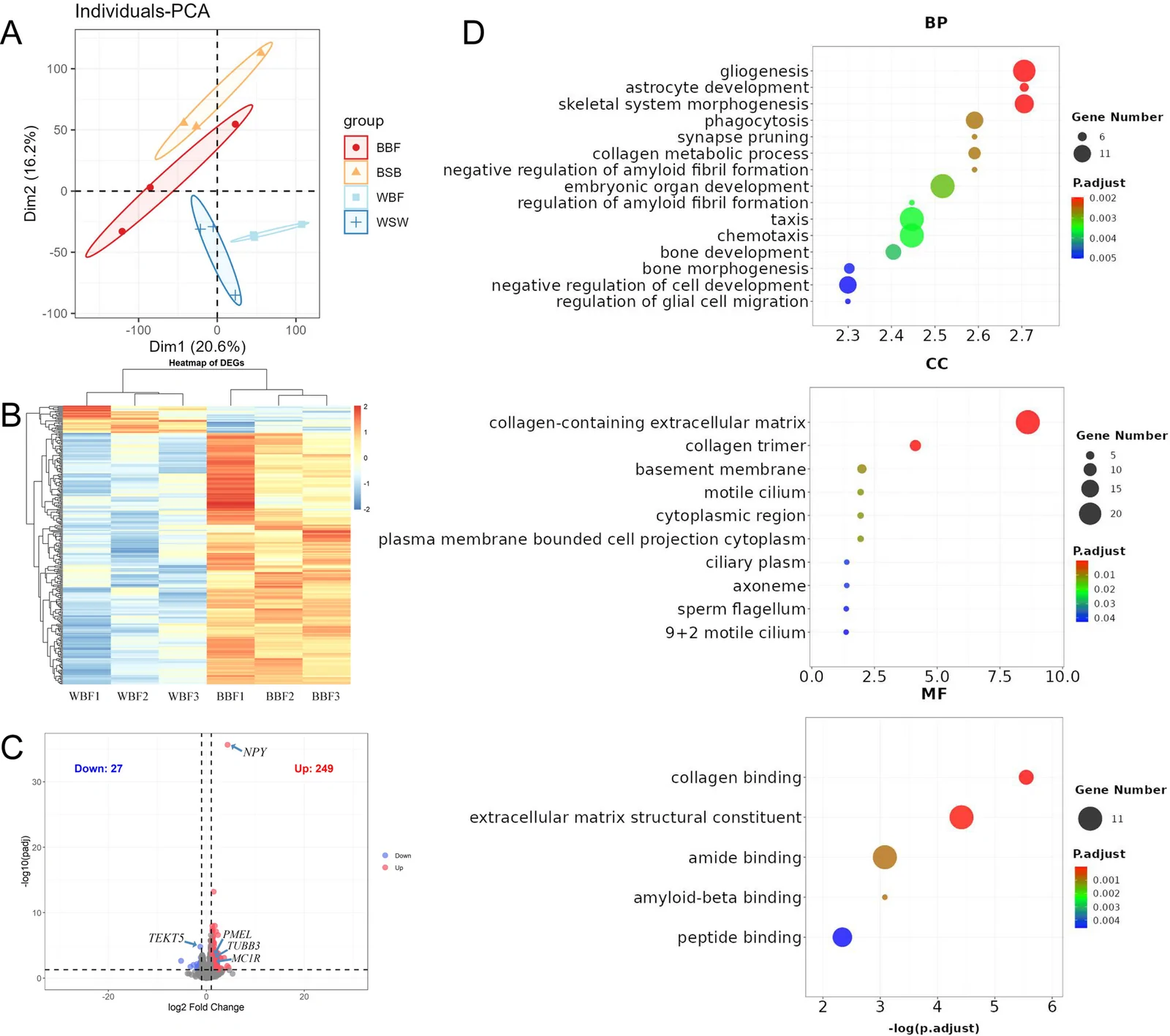
Transcriptomic differential expression analysis. **A** Principal component analysis (PCA) of RNA-seq data from 12 feather follicle samples, where each point represents one individual sample. Ellipses represent 95% confidence ellipses for each group. BSB: black feathers from solid black chickens (*n* = 3); BBF: black feathers from black-flecked chickens (*n* = 3); WBF: white feathers from black-flecked chickens (*n* = 3); WSW: white feathers from solid white chickens (*n* = 3). **B** Heatmap of differentially expressed genes (DEGs) between WBF and BBF. **C** Volcano plot showing the distribution of DEGs. **D** Gene Ontology (GO) enrichment analysis of DEGs. BP: Biological Process; CC: Cellular Component; MF: Molecular Function

To further investigate the regulatory mechanisms underlying phenotypic divergence in *I*/*i⁺* individuals, transcriptomic sequencing analysis was performed. PCA revealed distinct clustering patterns corresponding to feather pigmentation phenotypes rather than clustering based on the individuals (Fig. 3A). Specifically, white feathers from solid white chickens tended to cluster closer to white feathers from black-flecked chickens, whereas black feathers from solid black chickens showed greater similarity to black feathers from black-flecked chickens, despite the fact that the two feather types (WBF and BBF) were sampled from the same black-flecked individuals. These patterns suggest transcriptional differences associated with feather color.

DEGs among groups were identified using unified thresholds of | log₂(fold change) |≥ 1 and *Padj* < 0.05. We first compared BBF with WSW, and a total of 124 DEGs were detected, including 101 upregulated and 23 downregulated genes in BBF (Table S3). Notably, *MC1R* were among the significantly upregulated genes (Fig. S3A, B). Functional enrichment analysis of this comparison revealed significant enrichment in pigmentation-related biological processes, including “pigment biosynthetic process” “melanin biosynthetic process” and “melanin metabolic process” (Fig. S3C, Table S4), consistent with differences in pigmentation status between white and black feathers.

Rather than the BBF-WSW comparison, the transcriptomic differences between distinct feather colors within black-flecked individuals provide more direct insight into downstream regulatory mechanisms of pigmentation, as these samples were identical in genome. Therefore, DEGs between BBF and WBF were analyzed and a total of 276 DEGs were detected (Fig. 3B, C; Table S5). *PMEL*, *MC1R*, and *TUBB3* were significantly upregulated in black feathers relative to white feathers, highlighting their potential involvement in eumelanin deposition. GO enrichment analysis showed that these DEGs were significantly enriched in processes related to cell migration, tissue development, cell adhesion, and skeletal system development, including terms such as “regulation of cell migration” “skeletal system morphogenesis” and “bone development” (Fig. 3D, Table S6). These results suggest that intra-individual pigmentation differences may involve broader biological processes related to feather follicle development and microenvironmental remodeling, rather than being restricted to canonical melanogenesis pathways.

### Genes identified by integrative analysis of genomoic and transcriptomic data

**Fig. 4:**
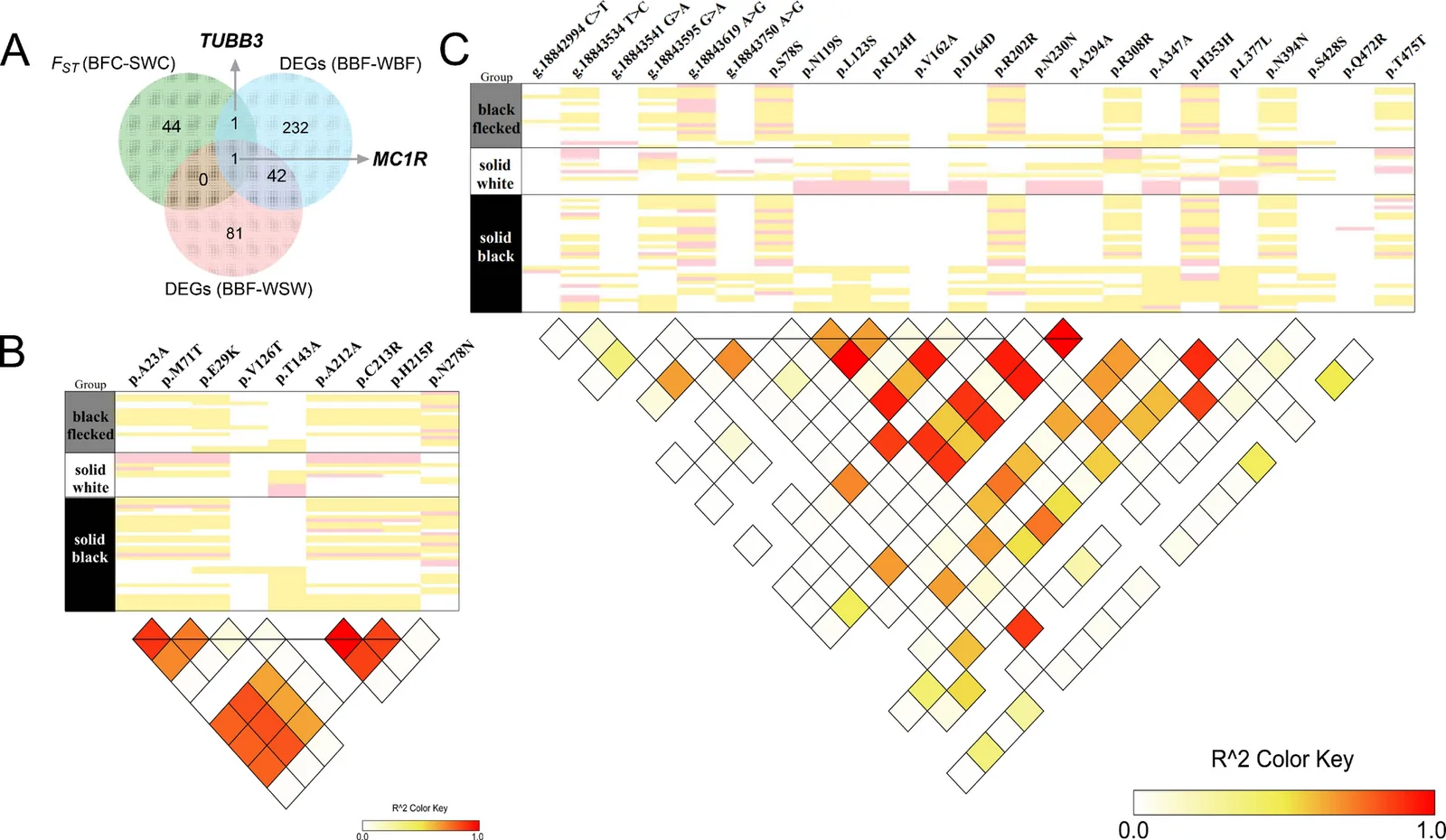
Results of integrative analysis of genomic and transcriptomic data. **A** Venn diagram of candidate genes identified from genomic and transcriptomic analyses. **B** LD plot and genotypic distribution at different mutation sites in the *MC1R* gene. **C** LD plot and genotypic distribution at different mutation sites in the *TUBB3* gene. Regarding the genotypic distribution, white cell indicates the homozygous wild-type genotype, yellow cell indicates the heterozygous genotype, and red cell indicates the homozygous mutant genotype. Variant nomenclature follows standard mutation annotation conventions. “g.” indicates genomic-level variants, whereas “p.” indicates protein-level amino acid substitutions

From the *ZF*_*ST*_ analysis between BFC and SWC, as well as the transcriptomic comparisons of BBF-WBF and BBF-WSW, *MC1R* was the only gene shared across all three analyses, whereas *TUBB3* is the only overlapping gene between *ZF*_*ST*_ and the BBF-WBF comparison but not with BBF-WSW. Accordingly, we focused subsequent analyses on *MC1R* and *TUBB3* (Fig. 4A).

In our samples, nine SNPs were identified in the coding region of *MC1R* through whole-genome sequencing (Fig. 4B). Genotypic comparison revealed that black-flecked and solid black chickens share high similarity at those SNP, whereas both are genetically distinct from solid white chickens. Among them, p.T143A (chr11:18,841,072, A > G) mutation exhibited a high allelic frequency in solid white chickens, a low frequency in black-flecked chickens (only with a small proportion being heterozygous), and was also predominantly wild-type in solid black chickens (Fig. 4B). Chi-square tests confirmed that the genotypic distribution at the p.T143A locus differed significantly among the three groups (Table 1). To further evaluate the potential function of this site, we performed comparative sequence analysis and found that the threonine residue at position 143 is highly conserved across vertebrate species, including birds and fish (Fig. S4).

**Table 1:** Genotypic distribution of p.T143A in *MC1R* among different groups

SNP	Genotype	Heterozygous at the *Dominant White* locus		Wild-type at the *Dominant White* locus	χ^2^ test *P*-value
		SWC	BFC	SBC	
p.T143A	AA	5 (0.38)	14 (0.78)	20 (0.61)	0.0059
	AG	4 (0.31)	4 (0.22)	13 (0.39)	
	GG	4 (0.31)	0	0	

- Genotypes were derived from the filtered VCF file based on WGS data.

- The numbers before the bracket are the observation count, and the values within the bracket are the genotypic frequency.

- “p.” indicates protein-level amino acid substitutions.

*TUBB3* is located 1,117 bp downstream of *MC1R* and encodes Tubulin Beta-3 Class III, a β-tubulin isoform expressed exclusively and constitutively in all neurons [44]. Among the identified coding mutations of *TUBB3*, a synonymous variant p.R202R (chr11:18,844,245, T > C) showed the most pronounced differentiation between black-flecked and solid white chickens: almost all solid white chickens were wild-type, whereas black-flecked and solid black chickens carried the mutation at high allelic frequencies (Fig. 4C, Table 2). These results suggest that the p.R202R variant is associated with the black-flecked phenotype in the genetic background of *I*/*i*^+^, whereas in the other genetic context, it is predominantly observed in solid black chickens.

**Table 2:** Genotypic distribution of p.R202R in *TUBB3* among different groups

SNP	Genotype	Heterozygous at the *Dominant White* locus		Wild-type at the *Dominant White* locus	χ^2^ test *P*-value
		SWC	BFC	SBC	
p.R202R	TT	11 (0.92)	2 (0.11)	9 (0.30)	0.0002
	TC	1 (0.08)	12 (0.67)	15 (0.50)	
	CC	0	4 (0.22)	6 (0.20)	

- Genotypes were derived from the filtered VCF file based on WGS data.

- The numbers before the bracket are the observation count, and the values within the bracket are the genotypic frequency.

- “p.” indicates protein-level amino acid substitutions.

The allelic frequencies of the two candidate mutations were checked in 68 public available WGS data of RJF (Table S1). As the results, the wild-type of p.T143A in *MC1R* and the mutant of p.R202R in *TUBB3* are dominant in RJFs (allelic frequencies are 0.963 and 0.923, respectively), suggesting that the alleles associated with BFC and SBC in our study originate from their ancestors. In other words, it is likely that the solid white plumage was artificial selected after the domestication of chicken, consistent with previous studies [45].

Given that *TUBB3* is the gene closest to *MC1R* (~ 1.1 kb away), we are uncertain about which mutation is functional or a linked marker, or whether both genes play roles in pigmentation. However, LD analysis revealed relatively weak LD between the *MC1R* and *TUBB3* regions, suggesting that these loci may not constitute a strongly linked haplotype block in our population (Fig. S5). To further examine the local chromatin organization surrounding this region, we referenced publicly available chicken Hi-C data from the FAANG project [41], which were generated from liver tissue. Notably, *MC1R* and *TUBB3* were located within the same topologically associating domain (TAD), suggesting potential chromatin-level proximity between these genes (Fig. S6).

### DEGs between feathers with the same color across different individuals

**Fig. 5:**
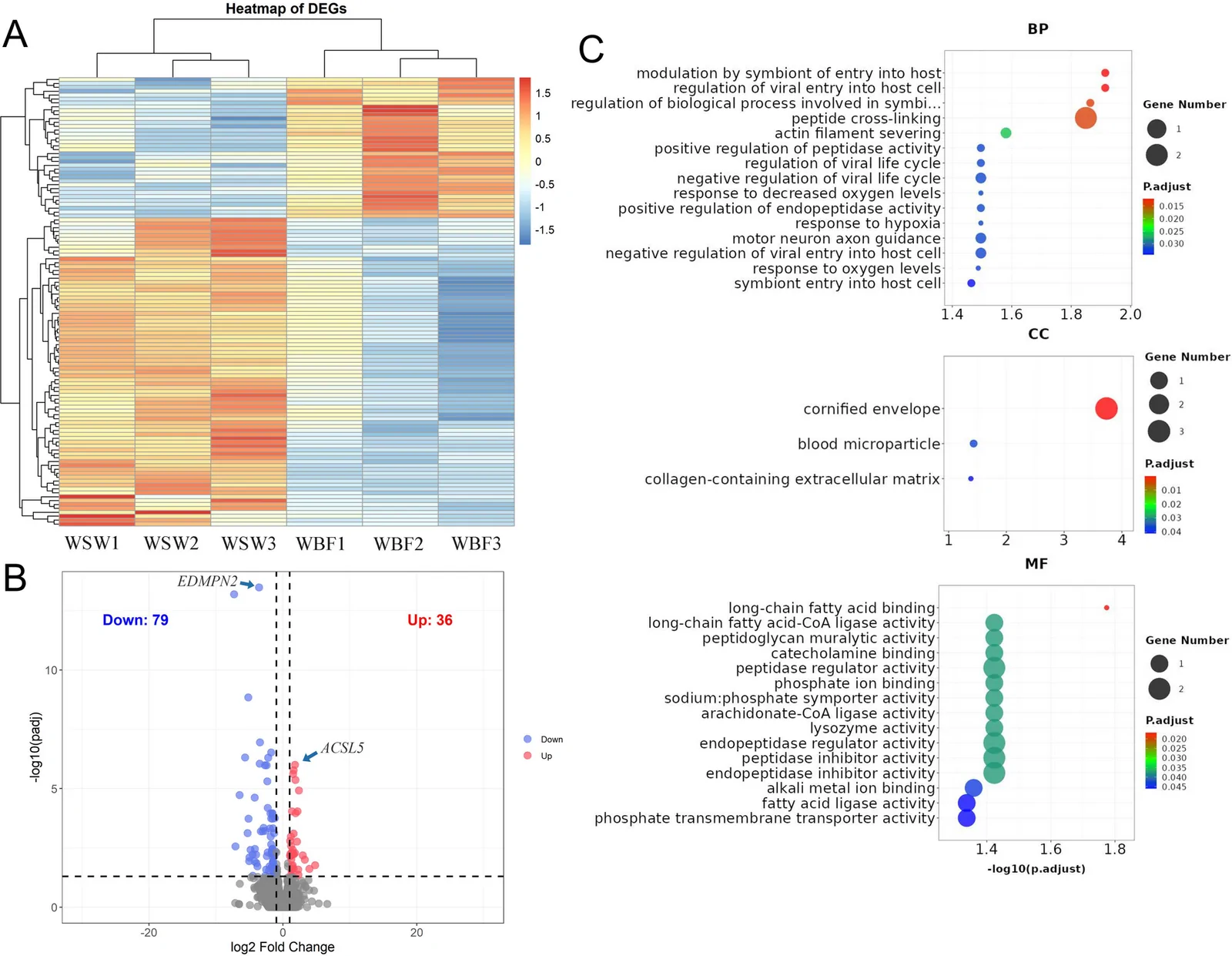
Transcriptomic differential expression analysis between WBF and WSW. **A** Heatmap of differentially expressed genes (DEGs) between white feathers from black-flecked chickens (WBF) and white feathers from solid white chickens (WSW). **B** Volcano plot showing the distribution of DEGs. **C** Gene Ontology (GO) enrichment analysis of DEGs. BP: Biological Process; CC: Cellular Component; MF: Molecular Function

We further compared WBF with WSW and BBF with BSB to characterize transcriptomic differences between feathers with similar pigmentation but different genetic backgrounds. In the comparison between WBF and WSW, a total of 115 differentially expressed genes were identified (Fig. 5A, B; Table S7), among which 36 genes were significantly upregulated and 79 genes were downregulated in WBF relative to WSW. Functional enrichment analysis indicated that these DEGs were mainly associated with biological processes related to cytoskeletal organization, extracellular matrix and structural organization, as well as metabolic and transport-related functions (Table S8). These results suggest that intra-pigment variation between WBF and WSW may be associated with differences in cellular structural organization and microenvironmental regulation within feather follicles. Notably, although no significant activation or suppression of melanogenesis-related pathways was detected at the GO level, key melanogenesis-related genes such as *PMEL* and *MLANA* showed decreased expression in BBF compared with BSB (Table S6; Fig. 5C). In contrast, *GPR143* exhibited increased expression in WBF compared with WSW. These results suggest that, although certain melanogenesis-related genes may show significant expression differences in feathers with similar pigmentation, such changes may not be sufficient to produce a significant biological effect.

In the comparison between BBF chickens and BSB chickens, a total of 219 DEGs were identified (Fig. S7A, B), including 162 upregulated and 57 DEGs in BBF relative to BSB (Table S9). GO enrichment analysis showed that these genes were mainly associated with cell migration, cell adhesion, chemokine/cytokine-mediated signaling, extracellular matrix organization, and skeletal system development (Table S10). These results suggest that the differences between BBF and BSB feathers may also be associated with remodeling of the extracellular microenvironment within feather follicles. Such differences are likely driven by alterations in intercellular communication, extracellular matrix organization, and tissue structural remodeling (Fig. S7C).

## Discussion

Different feather colors in chickens arise through distinct mechanisms, which confer different functions and benefits [1, 7–13]. Therefore, each feather color provides excellent materials to study how pigmentation and color formation processes are associated with selectively advantageous phenotypes and genotypes. Previous studies have shown that a 9 bp insertion in exon 10 of the *PMEL* gene in chickens, known as the *Dominant White* mutation, suppresses the production of eumelanin in feathers and skin [4]. However, our results suggest that the ability of *I*/*i*^*+*^ heterozygotes to completely suppress eumelanin deposition is influenced by additional genetic factors beyond the *PMEL* mutation. *ZF*_*ST*_ analysis identified the end of chromosome 11 as a key candidate region associated with the phenotypic divergence between solid white and black-flecked *I*/*i*^*+*^ individuals. This region includes genes such as *MC1R*, *SLC7A5*, *ATG7*, *CDK10*, *TUBB3*, *CDH1*, and *NQO1*. Among these, *MC1R* acts as a central regulator in melanin synthesis and widely participates in the regulation of melanin deposition [11, 12]. Mutations in *MC1R* are closely associated with human skin and hair color traits as well as coat color characteristics in various animals [46–48]. In ducks, comparisons between black and white feathers have also identified *MC1R* as a key gene [49]. *TUBB3* is expressed in human melanocytes and has been reported as a marker of neural crest–derived cells [50, 51]. *CDH1* mediates the anchoring and attachment of melanocytes to surrounding keratinocytes [52], and studies suggest it may play a potential role in the formation of white and red earlobe coloration in chicken [53]. *NQO1* has been found to enhance melanin production by regulating tyrosinase activity [54]. These genes, including *MC1R*, *TUBB3*, *CDH1*, and *NQO1*, are located in close genomic proximity within the same chromosomal region according to the reference genome (GRCg6a). Multiple mutations with epistatic effects may determine the simultaneous selection of several genes, resulting in enhanced eumelanin deposition in feathers [55]. Alternatively, a strongly positively selected mutation may cause neighboring mutations to be affected by genetic hitchhiking, resulting in similar selection signals [56]. Transcriptomic analysis further revealed that *MC1R* was significantly differentially expressed in the contrasts between BBF-WSW and BBF-WBF, respectively. The genotypic distributions of p.T143A site in *MC1R* showed significant differentiation between black-flecked and solid white chickens, indicating a strong association with phenotypic divergence in *Dominant White* heterozygotes. In addition, solid black chickens (carrying *E* allele) without the *I* allele did not show significant differentiation with black-flecked* I*/*i*^*+*^ individuals at *MC1R* locus. Thus, here is an interesting observation, because of the *E* allele, all the pheomelanin was replaced by the eumelanin [13], which was able to be inhibited by *I*. This leading to the fact that *E* contributed the solid white plumage in the *I*/*I* chickens. However, the *E* allele also allows black-flecked feathers to be expressed in *I*/*i*^*+*^ chickens.

Although *TUBB3* was not a differentially expressed gene in the BBF-WSW comparison, it is located in the candidate region on chromosome 11 in the *ZF*_*ST*_ analysis and in the BBF-WBF differential expressed gene set. Previous studies have shown that *TUBB3* expression decreases in senescent melanocytes and benign nevi, but increases in malignant melanoma [51]. This aligns with our observation in black-flecked individuals, where *TUBB3* expression was significantly lower in white feathers compared to black feathers. This pattern may suggest that, in an originally white-feathered background, the appearance of black pigment could reflect increased melanocyte activity associated with cytoskeletal remodeling regulated by *TUBB3* [50], although further experiments are required to confirm this hypothesis. Functionally, *TUBB3* is related to *MC1R*. Previous studies have shown that the expression level of *MC1R*-*TUBB3* chimeric mRNA can modulate the response of dermal melanocytes to ultraviolet radiation and may regulate melanocyte distribution through effects on cytoskeletal rearrangement [57]. Notably, the p.R202R site of *TUBB3* shows even a higher degree of differentiation between black-flecked and solid white chickens than the p.T143A site of *MC1R*. From a genomic perspective, *MC1R* (chr11:18,840,646–18,841,590) is located upstream of *TUBB3* (chr11:18,842,707–18,845,130), suggesting that this region may function cooperatively in feather color formation. Publicly available Hi-C data derived from chicken liver tissue showed that *MC1R* and *TUBB3* are located within the same topologically associating domain (TAD), suggesting potential chromatin-level proximity between these genes. However, because these Hi-C data were not generated from feather follicle tissues, their relevance to pigmentation-specific regulation should be interpreted cautiously. Furthermore, same as *MC1R*, black-flecked and solid black chickens show high similarity in genotypes at the *TUBB3* mutation site, while both differ genetically from white-feathered chickens. Therefore, *TUBB3* may also participate in the incomplete suppression of eumelanin deposition in *I*/*i*^*+*^ individuals. Kinoshita et al. (2014) reported *mo*^*w*^ mutation in chicken and identified the causal mutation as Cys244Phe in *EDNRB2* gene [58]. The phenotype of *mo*^*w*^ chicken is similar to the black-flecked phenotype observed in our study, thus we further examined genomic variations within the *EDNRB2* region. However, *EDNRB2* was not located within the differentiated genomic regions identified by *ZF*_*ST*_ analysis, and no *EDNRB2* mutations with obvious allelic frequency differentiation between solid white and black-flecked *I*/*i*^*+*^ individuals was observed. These results suggest that the black-flecked phenotype observed in this study may involve genetic mechanisms distinct from *mo*^*w*^ chickens.

In addition, the black and white feathers of black-flecked chickens in this study were from the same individual, so theoretically, the occurrence of two different feather colors may be influenced by external environmental factors or the local molecular microenvironment. In the BBF-WBF group, the uniquely differentially expressed genes were significantly enriched in biological processes related to tissue development, cell migration, skeletal system morphogenesis, and other developmental regulatory processes. These processes have previously been reported to be associated with pigment synthesis and feather development [59, 60]. The connection between glial cells and melanocytes mainly lies in their common origin from neural crest cells (NCCs), and both may respond to similar signals during differentiation. Studies have shown that epidermal growth factor (EGF) induces NCCs to differentiate into neurons and melanocytes, whereas fibroblast growth factor 2 (FGF2) promotes NCC differentiation into glial cells [61]. In the presence of both EGF and FGF2, neuronal differentiation is predominant [62]. Therefore, we hypothesize that under the influence of the activating mutation in *MC1R*-*TUBB3*, black-flecked individuals, although carrying* I* allele, have feather follicles throughout the body with the potential to synthesize eumelanin. However, only a small proportion of feathers successfully displayed eumelanin deposition, possibly because some unknown factors direct most melanocyte precursor cells toward glial cell differentiation, thereby losing the ability to transfer melanin to the feathers. Consistent with this hypothesis, several melanogenesis-related genes, including *TYR*, *TYRP1*, *GPR143*, *MLANA*, *SLC45A2*, and *RAB38*, did not show significant differential expression between BBF and WBF (Fig. S8, Table S3-S6) [63, 64]. We further examined the expression patterns of these genes across different feather types and found that genes such as *TYR*, *GPR143*, and *MLANA* showed relatively low expression levels in WSW, whereas their expression levels in WBF were closer to those observed in BBF (higher than those in WSW, although some genes did not reach statistical significance). However, these feathers still exhibited a white phenotype. These observations suggest that the phenotypic differences in melanin deposition may not necessarily arise from significant transcriptional changes in core melanogenesis genes, but are more likely attributable to altered cellular responses to upstream signaling mechanisms and regulatory environments. In addition, we found that *NPY* was the most significantly differentially expressed gene in the BBF–WBF comparison, and was also significantly upregulated in the BBF–WSW comparison. According to our data, *NPY* expression showed an overall decreasing trend from BSB to BBF, WBF, and WSW (Fig. S9, Table S3-S6), although not all pairwise differences were statistically significant. Overall, feathers with higher melanin deposition tended to exhibit higher *NPY* expression levels. *NPY* encodes neuropeptide Y, which is mainly highly expressed in the nervous system [65]. Neuropeptides are involved in regulating the migration, differentiation, and function of neural crest-derived cells, which can result in melanocytes [66]. Previous studies suggest that *NPY* may indirectly influence the melanocortin system by regulating AGRP (agouti-related peptide), but its direct role in melanin synthesis requires further investigation [67]. Previous studies have shown that the *I* allele impairs *PMEL* function and disrupts normal melanosome maturation, resulting in markedly reduced eumelanin deposition in feathers [4–6]. In this context, genes such as *MC1R*, *TUBB3*, and *NPY*, may compensate the effect of *I* on eumelanin synthesis and deposition, allowing a small portion of black feathers to form on a predominantly white background.

Furthermore, we found that a total of 43 genes, including *MC1R*, were significantly upregulated in both BBF-WSW and BBF-WBF groups. GO enrichment analysis of these 43 genes showed that the most significant term was response to organic cyclic compounds, which include aromatic compounds, steroids, terpenes, and furans, and are related to gene expression changes, metabolic regulation, and signal transduction [68, 69]. Notably, this GO term reflects cellular response states rather than the metabolic processing of these compounds themselves. Tyrosine and its metabolites, such as dopa and dopaquinone, which are required for melanin synthesis, are organic cyclic compounds [70]. In addition, cyclic adenosine monophosphate (cAMP), a key second messenger downstream of *MC1R* signaling, also belongs to organic cyclic compounds and plays a central role in regulating melanogenesis through the cAMP–PKA pathway [63]. Therefore, our results suggest that the observed transcriptional changes may reflect altered cellular responsiveness to signaling molecules associated with melanogenesis, rather than direct modulation of DOPA/cAMP metabolic pathways. Such changes in responsiveness may contribute to variation in melanosome activity, pigment deposition, and ultimately BBF plumage pattern formation, as previously reported in other vertebrate pigmentation systems [71–74].

Finally, we analyzed transcriptional differences in individuals with the same feather color phenotype but different genetic backgrounds, i.e., BBF-BSB and WBF-WSW comparisons. Consistent with the observations from comparisons among distinct feather color phenotypes, In both comparisons, pathways related to extracellular matrix organization, cell adhesion, and tissue structural remodeling were significantly enriched, suggesting that these processes may indirectly affect pigmentations by masking the effects of *I* or *E*. Despite exhibiting similar downstream melanogenesis pathways which are directly responsible for the pigmentation, individuals with different genetic backgrounds (*I* or *E*) displayed distinct upstream molecular signatures associated with feather follicle development and extracellular microenvironment remodeling. These results suggest that feather color, again is not only mediated by the expression levels of classical pigment-related genes, but may also involve interactions among local tissue structures, intercellular communication, and metabolic states within feather follicles.

## Conclusions

This study investigated the genetic basis underlying phenotypic heterogeneity in *Dominant White* heterozygous (*I*/*i*^+^) chickens. Genomic and transcriptomic analyses identified *MC1R* and *TUBB3*, which showed strong association with pigmentation divergence within *I*/*i*^*+*^ individuals. Overall, our findings suggest that the black-flecked plumage observed in *I*/*i*^*+*^ chickens may reflect localized heterogeneity in feather follicle regulatory environments, resulting in spatially variable eumelanin deposition. This phenomenon likely involves the combined effects of underlying genetic determinants, tissue microenvironment, and cellular responsiveness to upstream regulatory signals.

## Supplementary Information

Supplementary Material 1.

## Data Availability

The raw sequencing data have been deposited in the Genome Sequence Archive (https://ngdc.cncb.ac.cn/search/all?&q=PRJCA066542) under the accession number PRJCA066542. Detailed information for all samples used in this study, including sample group assignments, sequencing data types, and corresponding accession numbers, is provided in Supplementary Table S11.
