# Supplementary material for: Genomic and transcriptomic analysis suggests potential regulatory relationships among MC1R, TUBB3, and PMEL associated with black-flecked plumage in chickens
Source: BMC Genomics. 2026 Jun 29;27:764. doi: 10.1186/s12864-026-13107-w (PMC13584571; doi:10.1186/s12864-026-13107-w)
Supplement: Supplementary file 1 [file 12864_2026_13107_MOESM1_ESM.zip › Supplementary figure.docx]

Fig. S1. Representative PCR-RFLP genotyping results for the I locus M indicates the DNA molecular weight marker (500 bp DNA ladder; bands from top to bottom correspond to 500, 400, 300, 250, 200, 150, 100, and 50 bp). Lanes show digestion patterns corresponding to different genotypes as described in the main text.

Fig. S2. Genome-wide *ZF_ST_* analysis of individuals with different feather color phenotypes A: Manhattan plot of *ZF_ST_* analysis between solid white chicken (SWC) and solid black chicken (SBC). B: Manhattan plot of *ZF_ST_* analysis between black-flecked chickens (BFC) and SBC. The first gray threshold line represents the top 0.1% *ZF_ST_* values, while the second black threshold line represents the top 1% *ZF_ST_* values.

Fig. S3. Transcriptomic differential expression analysis between WSW and BBF A: Heatmap of differentially expressed genes (DEGs) between white feathers from solid white chickens (WSW) and black feathers from black-flecked chickens (BBF). B: Volcano plot showing the distribution of DEGs. C: Gene Ontology (GO) enrichment analysis of DEGs. BP: Biological Process; CC: Cellular Component; MF: Molecular Function.

Fig. S4. Conservation analysis and structural modeling of *MC1R* p.T143A A. Multiple sequence alignment of amino acid residues at and surrounding position 143 of *MC1R* across representative vertebrate species. B. Predicted protein structure of the wild-type *MC1R*, with the amino acid at position 143 indicated. C. Predicted protein structure of the mutant *MC1R* (T143A), showing the substitution at position 143.

Fig. S5. Linkage disequilibrium (LD) pattern between the MC1R and TUBB3 regions.

Fig. S6. Public Hi-C interaction map of the MC1R-TUBB3 genomic region in chicken liver tissue.

Fig. S7. Transcriptomic differential expression analysis between BBF and BSB A: Heatmap of differentially expressed genes (DEGs) between black feathers from black-flecked chickens (BBF) and black feathers from solid black chickens (BSB). B: Volcano plot showing the distribution of DEGs. C: Gene Ontology (GO) enrichment analysis of DEGs. BP: Biological Process; CC: Cellular Component; MF: Molecular Function.

Fig. S8. Expression levels of *TYR*, *TYRP1*, *GPR143*, *MLANA*, *SLC45A2*, *RAB38*, in three feather color groups based on FPKM values BBF: black feathers from black-flecked chickens (n = 3); WBF: white feathers from black-flecked chickens (n = 3); WSW: white feathers from solid white chickens (n = 3). Values are presented as mean ± standard error (SE). Statistical significance was evaluated based on adjusted p-values (padj) obtained from DESeq2 differential expression analysis.

Fig. S9. Expression levels of NPY in four feather color groups based on FPKM values BSB: black feathers from solid black chickens (n = 3); BBF: black feathers from black-flecked chickens (n = 3); WBF: white feathers from black-flecked chickens (n = 3); WSW: white feathers from solid white chickens (n = 3). Values are presented as mean ± standard error (SE). Statistical significance was evaluated based on adjusted p-values (padj) obtained from DESeq2 differential expression analysis.
